# Fulminant Pyoderma Gangrenosum After Outpatient Knee Arthroscopy

**DOI:** 10.5435/JAAOSGlobal-D-21-00006

**Published:** 2021-08-20

**Authors:** Taylor Bates, Andrew J. Sheean, Erica Kao, Justin P. Bandino, Thomas B. Lynch, Dustin Lybeck

**Affiliations:** From the Department of Orthopaedic Surgery(Dr. Bates, Dr. Sheean, Dr. Lynch, Dr. Lybeck); Department of Pathology (Dr. Kao); and Department of Dermatology (Dr. Bandino), San Antonio Military Medical Center, Fort Sam Houston, TX.

## Abstract

Pyoderma gangrenosum is an immunologic, ulcerative cutaneous condition often associated with systemic disease and frequently precipitated by trauma. It is noninfectious, but the inflammatory assault can resemble a malignant infection such as necrotizing fasciitis. Despite its clinical resemblance to infection, surgical débridement worsens the condition and may remove morphologic clues to the true disease, thus creating a vicious cycle of surgical débridements and disease progression. Furthermore, diagnostic histopathologic and laboratory features are nonspecific, requiring exclusion of other processes. Therefore, appropriate nonsurgical treatment and immunosuppression are commonly delayed, often at a significant cost to the patient. We present a case of pyoderma gangrenosum occurring after outpatient knee arthroscopy that masqueraded as a postsurgical infection. We discuss the diagnostic approach and how a complex reconstruction involving cartilage restoration and soft-tissue coverage was achieved.

Pyoderma gangrenosum (PG) is an immunologic ulcerative cutaneous condition of an undetermined cause that is often precipitated by trauma. The incidence varies between studies with estimates typically ranging from 3 to 10 per million per year.^[Bibr R1][Bibr R2][Bibr R2][Bibr R4]^ The disease typically presents in the fourth through sixth decade of life and has a female predominance.^1,5-7^ It can be easily mistaken for an infectious process such as necrotizing fasciitis (NF) due to the rapidly progressing wound deterioration in the presence of elevated inflammatory markers and leukocytosis. In contrast to infectious processes, such as NF, surgical débridement promotes the malignant immunologic response, leading to further skin breakdown.

A high index of suspicion and the use of a multidisciplinary approach can help differentiate PG from other etiologies, such as a surgical site infection (SSI). Timely workup is paramount because a failure to differentiate the two processes—immunologic and infectious—will lead to misdiagnosis and detrimental interventions. Therefore, early involvement of infectious disease specialists and dermatologists when the diagnosis is in question can help determine whether serial débridements should be initiated or continued.

We present a novel case of PG masquerading as an SSI after knee arthroscopy that was successfully treated with a comprehensive, multidisciplinary approach focused on limb salvage. The purpose of this case report was to describe the clinical presentation and successful treatment of fulminant, multifocal PG after outpatient knee arthroscopy.

## Case Report

The patient was a 35-year-old male active-duty US Navy Sailor who developed a severe inflammatory reaction of the anterior knee, fever, elevated inflammatory markers, and markedly elevated white blood cell (WBC) count after an arthroscopic procedure. The initial arthroscopic surgery was done at an outside facility for the treatment of a medial femoral condyle (MFC) osteochondral lesion that required screw fixation. He has no history of systemic illness or autoimmune disorders, including inflammatory bowel disease (IBD) or inflammatory arthritis.

**Figure 1:**
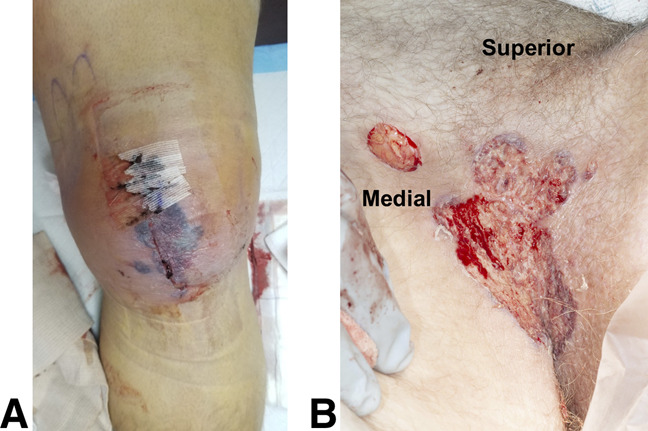
**A**, Clinical photograph after the initial irrigation and débridement demonstrating peri-incisional inflammation with violaceous to gunmetal gray color and poor postoperative healing. **B**, Ulcerative lesion of the left inguinal fold demonstrating punched-out serpiginous and violaceous to gunmetal gray border with a necrotic and cribriform center.

Eight days postoperatively, he presented to his local hospital with a febrile illness, edema, and erythema about the knee. His symptoms rapidly progressed to include pustules and ulcerations adjacent to his surgical incisions. Laboratory evaluation revealed elevated inflammatory markers and leukocytosis of 50,000 WBCs per μL. Broad-spectrum antibiotics (ie, vancomycin, Zosyn, and cefepime) and fluconazole were initiated before undergoing serial surgical débridements. Tissue cultures obtained during the time of initial débridement grew *Staphylococcus epidermidis*. However, cultures obtained during subsequent débridements failed to demonstrate the growth of an identifiable organism, and we think that the *S epidermidis* grown in the initial cultures were likely a contaminant. Remote to the wound breakdown at the surgical site (Figure 1, A), the patient developed painful, enlarging ulcerations in bilateral inguinal folds measuring approximately 1 and 5 cm that arose over the following 3 weeks (Figure 1, B). These ulcerations were similar in appearance to the wound involving the surgical site, exhibiting irregular, ovoid gray borders with necrotic-appearing centers.

**Figure 2:**
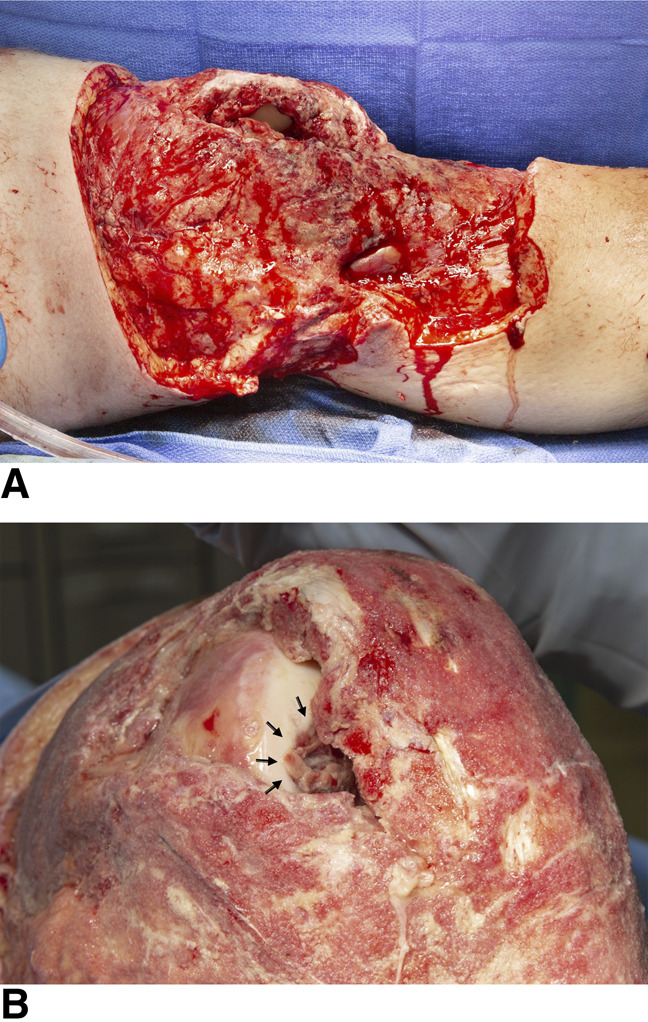
**A**, Intraoperative photograph demonstrating a large soft-tissue defect that includes the medial joint capsule of the left knee. **B**, Intraoperative photograph demonstrating the osteochondral defect of the medial femoral condyle (arrows) and joint capsule defect over the left knee.

**Figure 3:**
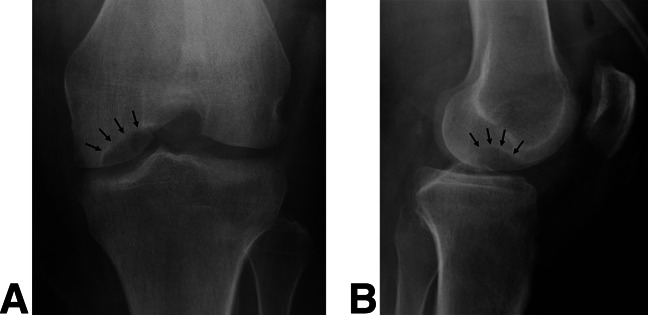
**A** and **B**, Orthogonal plain radiographs of the right knee demonstrating a corresponding, well-circumscribed lucency in the vicinity of the osteochondral lesion (arrows).

The patient was ultimately transferred to our tertiary referral center. On arrival, the soft-tissue defect about the knee was found to be nearly circumferential with full-thickness and subcutaneous tissue loss measuring approximately 30 × 20 cm. In addition, there was a 4 × 2 cm medial joint capsular defect, exposed MFC articular surface (Figure 2, A and B), and purulent tissue noted around the periphery of the wound. The underlying MFC osteochondral lesion measured 2.5 cm in diameter and was estimated to be approximately 25% uncontained on its inferolateral border, which corresponded with orthogonal radiographs of the affected knee and demonstrated a well-circumscribed lucency about the lateral aspect of the MFC (Figure 3, A and B).

**Figure 4:**
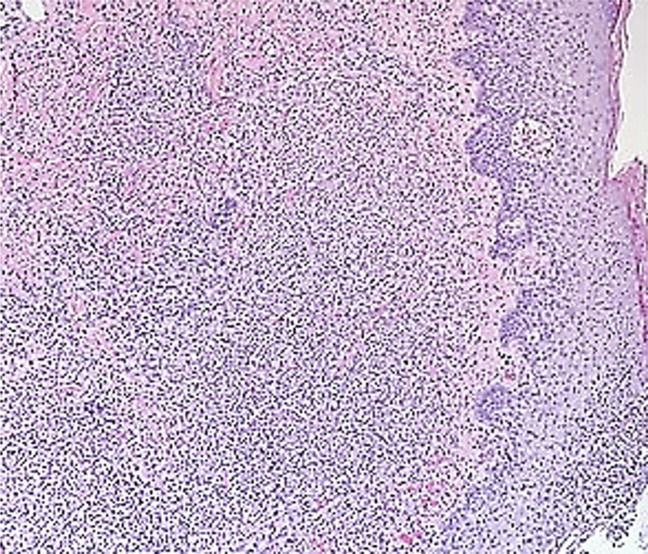
Photomicrograph of a lower power view from the edge of the ulcer demonstrating a dense, neutrophilic infiltrate in the dermis (H&E, original magnification, 200x).

Serial débridements and broad-spectrum antibiotics were continued while a comprehensive diagnostic workup was conducted by a multidisciplinary team comprising specialists in orthopaedic surgery, burn surgery, infectious disease, pathology, and dermatology. The differential diagnosis of an infectious etiology was broad and included entities such as NF, septic arthritis caused by culture-negative *S aureus*, fungal arthritis (ie, *Candida* species), nontuberculous mycobacterial arthritis, brucellosis, and gonococcal arthritis. Histologic specimens derived from the tissue taken during subsequent débridements demonstrated a neutrophilic dermatosis with greater than 100 polymorphonuclear neutrophils per high-powered field with no organisms identified (Figure 4). PG was considered based on his failure to improve with antimicrobial therapies, negative wound cultures, poor response to débridements , the clinical appearance, ulcerations of the groin, and histologic features on biopsy.

Given the preponderance of clinical evidence and the lack of improvement with surgical débridement and antimicrobial therapy, the diagnosis of PG was considered likely by the multidisciplinary team. Because no specific testing was available for PG, the team began treatment with close observation. Systemic intravenous (IV) pulse corticosteroids (1,000 mg methylprednisolone per day for 5 days) were initiated followed by a rapid clinical improvement. After a course of IV methylprednisolone, he was transitioned to oral prednisone 1 mg/kg/d, followed by a slow taper over the following months. Débridements and negative pressure dressing changes were conducted every 2 to 3 days in preparation for definitive soft-tissue coverage. Corticosteroid prophylaxis, which included calcium and vitamin D supplementation, was begun given the long course of corticosteroids.

After 2 weeks of steroid treatment and a total of nine débridements, a definitive reconstructive procedure was done. A 25 mm in diameter and 10-mm-thick osteochondral allograft was used to fill the defect, and a medial gastrocnemius muscle flap and skin grafting were used for soft-tissue coverage of the joint. The use of a gastrocnemius flap was based on the location requiring coverage and was done by the orthopaedic surgery service, which included tumor and sports specialists. This step was followed with split-thickness skin grafting done by the burn unit's surgical team.

**Figure 5:**
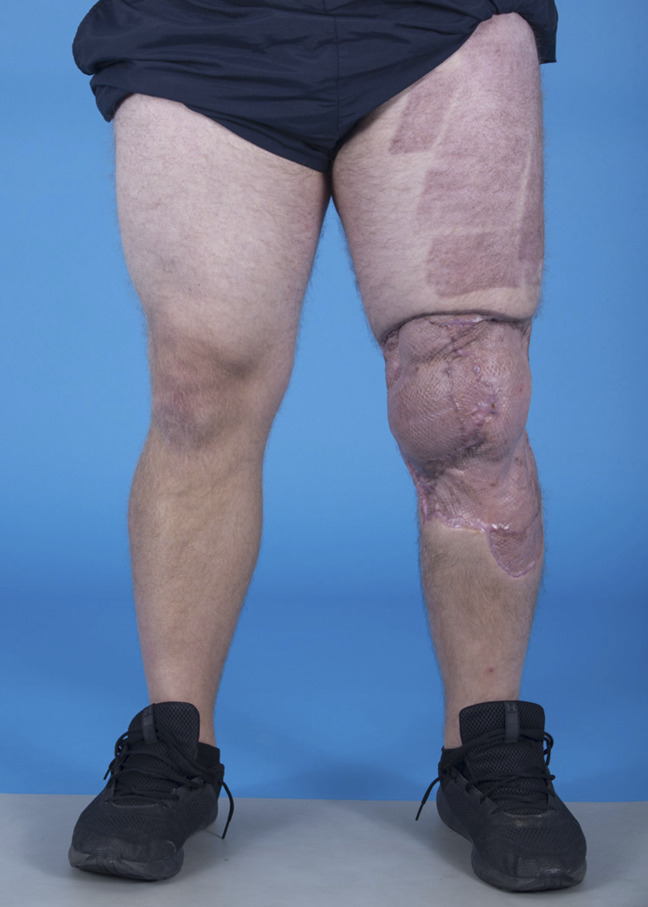
Clinical photograph 4 months postoperatively demonstrating complete healing of the split-thickness skin grafts.

**Figures 6:**
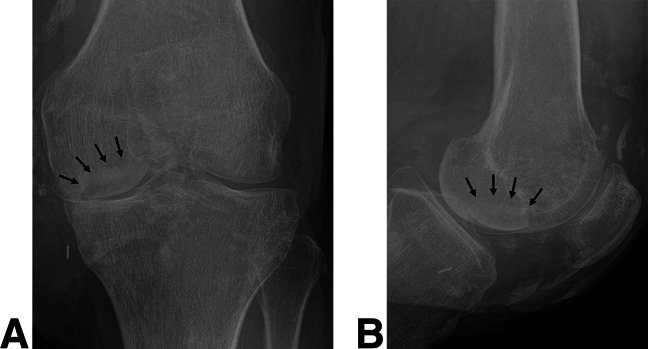
**A** and **B**, Orthogonal radiographs 12 weeks postoperatively demonstrating appropriate placement of the bone plug in the lateral aspect of the medial femoral condyle with congruence of the subchondral bone and osseous incorporation at the osteochondral allograft recipient site interface (arrows).

His wounds healed without complication, and no further procedures for soft-tissue coverage were required (Figure 5). Postoperative imaging at 12 weeks demonstrated osseous incorporation of the osteochondral allograft at the recipient site (Figure 6, A and B). One year postoperatively, his knee range of motion (ROM) was 0 to 130°, his pain level rated by the visual analog scale was 2, his International Knee Documentation Committee score was 77/87 (88.5%), and the Lysholm Knee Score was 79/100 (79%). The underlying cause of disease in this patient remains unknown, although surgical trauma is thought to be the initiating factor that led to an exaggerated inflammatory response, likely through upregulation of neutrophils. He has remained on active-duty status in the US Navy.

## Discussion

This case report presents an extremely rare postoperative condition that should be considered early in the workup for an SSI that is not responding to standard treatments. In addition, this report describes our experience managing an osteochondral lesion in the setting of a large soft-tissue defect and immunosuppressive therapy for the treatment of PG.

PG is a noninfectious neutrophilic dermatosis commonly misdiagnosed as an aggressive skin infection. The condition exhibits pathergy, meaning that skin lesions or ulcers can arise after minor trauma. Thus, PG is exacerbated by surgical débridement and is often associated with skip lesions, such as those arising at IV sites.^8^ Pathergy testing can be done, although was not used in this case. Elevated inflammatory markers and leukocytosis are common given the degree of inflammation and reinforce the concern for a serious infection.

The pathogenesis of PG is not fully understood. Histologic features of PG lesions include a diffuse dermal infiltration of polymorphonuclear neutrophils with leukocytoclasia without microorganisms (Figure 4). Aberrant neutrophil trafficking and subsequent release of cytokines, such as interleukin-8, seem to play a significant role in the development of the disease.^9^ A clinical review of published cases has found the mean time to onset of postoperative PG to be 7 days (range, 2 to 30 days), with a mean time to diagnosis of 15 days.^1^

Clinical features of PG typically include single or multiple small red tender nodules or pustules that rapidly progress to cribriform and necrotic ulcers with irregular, circumscribed violaceous and gunmetal gray undermined and overhanging borders that expand centrifugally.^10-12^ Patients usually present with involvement of the lower extremities or trunk and often have concomitant inflammatory or immunologic disorders.^7^ Systemic diseases are found in up to 50% or more of cases and therefore warrant a multidisciplinary workup after diagnosis of PG for other occult illnesses, such as IBD and malignancy.^3^ A known history of IBD, inflammatory arthritis, or a hematologic disorder (eg, IgA monoclonal gammopathy, acute myelogenous leukemia, and myelodysplasia) serves as a historical clue to aid in the delineation of PG versus SSI.

PG is a diagnosis of exclusion with no specific histopathologic or laboratory findings for diagnostic confirmation. Because of its nonspecific pathology findings, members of the treatment team must maintain a high suspicion while ruling out other potentially life-threatening or limb-threatening etiologies. Because the skin lesions caused by PG may resemble a variety of other conditions known to cause necrotizing ulcerations, the differential diagnosis should consider both infectious and noninfectious causes. Entities that may present similarly include, but are not limited to, necrotizing soft-tissue infections, brown recluse spider bites (necrotizing arachnidism), deep mycoses (eg, blastomycosis and sporotrichosis), vasculopathy, medium-vessel vasculitis, krokodil-induced skin necrosis, and warfarin-induced skin necrosis.^13,14,15,16^

Laboratory findings are often similar to infection, such as elevated inflammatory markers and WBC. The WBC in this report reached as high as 50,000 per μL, and his count remained markedly elevated until proper treatment with steroids was initiated. Histological evaluation is nonspecific but can be helpful to rule out other suspected causes. Certain clinical, laboratory, radiographic, and histological features can be used to help differentiate between the two diagnoses (Table 1).^8^

**Table 1:** Comparison of clinicopathologic features of PG vs. NF

	PG	NF
Time for lesions to develop	Days (7 d mean)^1^	Hours
Common comorbidities	Autoimmune diseases (ie, IBD) and malignancy	Immune suppression, malignancy, diabetes mellitus, obesity, bacterial inoculation (ie, IV drug use), renal failure, vascular disease, and chronic disease
Skip lesions	Often present	Rare
Pathergy	Present	Absent
Laboratory examination	Leukocytosis with elevated ESR and CRP	Same as PG
Wound cultures	Sterile unless contaminated	Variable, often polymicrobial, gas-forming bacteria
Imaging (radiograph and MRI)	Soft-tissue edema without gas formation along fascial planes	Soft-tissue edema with gas formation
Histology	Dense PMN infiltrate with absent microorganisms	Necrotic fascial layer with abundant microorganisms and PMNs
Treatment	Nonsurgical wound management and systemic immunosuppression	Aggressive débridement and broad-spectrum antibiotic therapy

CRP = C-reactive protein, ESR = erythrocyte sedimentation rate, IBD = inflammatory bowel disease, MRI = magnetic resonance imaging, NF = necrotizing fasciitis, PG = pyoderma gangrenosum, and PMN = polymorphonuclear neutrophil

The treatment of PG typically consists of a combination of systemic and topical agents with wound care using nonadherent dressings that promote a moist environment, taking care not to induce additional trauma.^17^ Corticosteroids are often the treatment of choice, but biologics, mycophenolate mofetil, minocycline, cyclosporine, IV immune globulin, or thalidomide may also be used.^6^ Although these medications may delay healing and predispose to infection, they calm the destructive inflammatory storm and ultimately help with wound healing. Continuation of immunosuppressive therapies during surgical procedures should be considered to help mitigate the rise of new lesions.

We report a case of PG masquerading as a postsurgical infection after knee arthroscopy that demonstrates the role of a multidisciplinary approach. With this approach, we achieved a good outcome in a patient who underwent allografting for an osteochondral defect with successful osseous integration, a rotational muscle flap for soft-tissue coverage, and resolution of skin lesions with immunosuppressive treatment. Orthopaedic surgeons should consider this diagnosis when a suspected SSI fails to respond to traditional medical and surgical treatments (Table 2).

**Table 2:** Summary of Recommendations

Recommendations
• Consider early involvement of infectious disease and dermatology experts in patients with suspected surgical site and necrotizing soft-tissue infections that fail to respond to traditional treatments • Screen each patient for a personal or family history of autoimmune disorders or symptoms of autoimmune disease and malignancy • Seek help from a gastroenterology specialist to rule out concomitant disease, such as IBD • Consider initiating corticosteroid prophylaxis with calcium and vitamin D supplementation • Avoid tissue trauma that could result in the propagation of pathergic disease (ie, unnecessary intravenous sticks)

IBD = inflammatory bowel disease

## Conclusion

PG is an inflammatory ulcerative disease often precipitated by trauma that can masquerade as an SSI. Despite being well recognized and extensively reported in the medical literature, the diagnosis is often delayed until surgical and medical management addressing an infection fails. The diseases' pathergic response magnifies the misdiagnosis with a potentially significant increase in morbidity and disfigurement. PG should remain as a differential diagnosis when treating SSIs, particularly when progression occurs despite treatment. Consultation with a multidisciplinary team that includes dermatologists and infectious disease specialists is recommended.
